# Survival of Women Previously Diagnosed of Melanoma with Subsequent Pregnancy: A Systematic Review and Meta-Analysis and a Single-Center Experience

**DOI:** 10.3390/jcm11010083

**Published:** 2021-12-24

**Authors:** Nieves Martínez-Campayo, Sabela Paradela de la Morena, Sonia Pértega-Díaz, Luisa Iglesias Pena, Pia Vihinen, Kalle Mattila, Marko B. Lens, Antonio Tejera-Vaquerizo, Eduardo Fonseca

**Affiliations:** 1 Department of Dermatology, University Hospital of A Coruña, 15006 Corunna, Spain; sabelaymarina@yahoo.es (S.P.d.l.M.); luipe96@gmail.com (L.I.P.); Eduardo.Fonseca.Capdevila@sergas.es (E.F.); 2 Department of Research, University Hospital of A Coruña, 15006 Corunna, Spain; Sonia.Pertega.Diaz@sergas.es; 3 FICAN West Cancer Centre and Department of Oncology, Turku University Hospital and University of Turku, 20521 Turku, Finland; Pia.Vihinen@tyks.fi (P.V.); Kalle.Mattila@tyks.fi (K.M.); 4 Genetic Epidemiology Division, Cancer Research UK, St. James’s University Hospital, Beckett St., Leeds LS9 7TF, UK; markolens@aol.com; 5 Department of Dermatology, Dermatologic Institute GlobalDerm, Palma del Río, 14700 Cordova, Spain; antoniotejera@aedv.es; 6 Unit of Cutaneous Oncology, San Juan de Dios Hospital, 14012 Cordova, Spain

**Keywords:** melanoma, pregnancy, prognosis, meta-analysis, systematic review, survival

## Abstract

Melanoma incidence has increased over the last few decades. How the prognosis of a previously diagnosed melanoma may be affected by a woman’s subsequent pregnancy has been debated in the literature since the 1950s, and the outcomes are essential to women who are melanoma survivors in their childbearing years. The main objective of this systematic review is to improve the understanding of whether the course of melanoma in a woman may be altered by a subsequent pregnancy and to help clinicians’ diagnosis. Eligible studies for the systematic review were clinical trials, observational cohort studies and case-control studies that compared prognosis outcomes for non-pregnant patients with melanoma, or pregnant before melanoma diagnosis, versus pregnant patients after a diagnosis of melanoma. The search strategy yielded 1101 articles, of which 4 met the inclusion criteria for the systematic review. All the studies were retrospective non-randomised cohorts with patients with melanomas diagnosed before pregnancy. According to our findings, a subsequent pregnancy was not a significant influence on the outcome of a previous melanoma. However, given the small number of identified studies and the heterogeneous data included, it is recommended to approach these patients with caution, and counselling should be given by known prognostic factors. We also reviewed the medical records of 84 patients of childbearing age (35.8 ± 6.3 years, range 21–45 years) who were diagnosed with cutaneous invasive melanoma in our hospital between 2008 and 2018 (N = 724). Of these, 11 (13.1%) had a pregnancy after melanoma diagnosis (age at pregnancy: 35.6 ± 6.3 years). No statistical differences in outcome were detected.

## 1. Introduction

Melanoma incidence has experienced a significant increase over the last few decades [1]. Europe and North America have a relatively flat age melanoma distribution curve so that it is relatively common in young adults. The thickness and also the survival are better during childbearing years than at other times of life [2]. Nowadays, the desire to conceive after a melanoma diagnosis is increasing because of delayed childbearing. Melanoma accounts for 31% of all malignant tumours diagnosed in pregnant women [3,4]. How pregnancy may affect a malignant disease has been discussed for years. Historically, elevated oestrogen levels have been hypothesised to increase the biologic aggressiveness of cancer cells regarded as hormone-dependent, such as ovarian and breast cancer and malignant melanoma [5,6,7,8].

How the prognosis of a previously diagnosed melanoma may be affected by a woman’s subsequent pregnancy has been debated in the literature since the 1950s, and the outcomes are essential to women who are melanoma survivors in their childbearing years.

In 2015, Byrom et al. [9] reported a systematic review and a meta-analysis to answer the previous questions. The results showed that current evidence does not support the hypothesis that pregnancy after successful treatment of melanoma worsens prognosis. However, relevant data were sparse, suggesting that a precautionary approach is warranted regarding childbearing advice to melanoma survivors [9]. Patients can be told that the relationship between melanoma and pregnancy is not fully understood, and the decision to conceive should incorporate the patient´s medical history and personal preferences [10].

The main objective of this study is to determine whether pregnancy after a melanoma diagnosis worsens the prognoses. We decided to perform a new systematic review including the most recently published literature, optimising the search parameters of the above-mentioned review. We aim to improve the understanding of whether the prognosis of melanoma in a woman may be altered by a subsequent pregnancy and to provide advice regarding family planning for clinicians treating women diagnosed with melanoma during their childbearing years.

## 2. Materials and Methods

Prognostic study. A retrospective observational study following Strengthening the Reporting of Observational Studies in Epidemiology guidelines [11] was designed. Patients of childbearing age (<45 years of age), who were diagnosed with cutaneous invasive melanoma in our hospital between 2008 and 2018, were included. We compared outcome measures of women with melanoma diagnosed prior to pregnancy with those who were not pregnant after the melanoma diagnosis. Overall, disease-free and melanoma-specific survival were estimated using Kaplan-Meier survival curves. Differences between pregnant and non-pregnant women were analysed with the log-rank test. Statistical analysis was performed with software SPSS 25.0 for Windows (IBM Corp., Armonk, NY, USA).

The systematic review was carried out following PRISMA (Preferred Reporting Items for Systematic Reviews and Meta-Analysis) guidelines and registered in PROSPERO (International Prospective Register of Systematic Reviews), CRD42020172131.

### 2.1. Search Strategy

The Cochrane Database, EMBASE and PubMed were searched using the attached strategy (Appendix A). The search was performed in November 2019 and was limited to studies in humans published in English and Spanish in the past twenty years.

The main search and the screening of titles and abstracts were completed independently by two reviewers (NML and LIP). Conflict studies for the previous reviewers (NML and LIP) were evaluated by two different reviewers (SPM and EF). Studies of female patients with melanoma meeting the following criteria were included in the analysis: (a) existence of two groups of patients with melanoma: pregnancy after melanoma and control or equivalent; (b) any outcome related to survival was reported in the study with the intention of comparing the included groups.

### 2.2. Selection of Relevant Studies

Eligible studies for the systematic review were clinical trials, observational population-, clinical- or hospital-based cohort studies and case-control studies that compared prognosis outcomes for non-pregnant patients with melanoma or those pregnant before a melanoma diagnosis versus pregnant patients after a melanoma diagnosis. Case series, case reports, reviews, abstracts, letters to the editor and cross-sectional studies were not eligible.

### 2.3. Data Extraction

Information from each study was extracted by two reviewers (NML and LIP) using a standardised data extraction form. Data were coded, entered in the computer and analysed using SPSS Statistics. General characteristics of the study and outcomes for both intervention and control groups were recorded.

The primary outcome measure was the overall survival (OS) of pregnant female patients after melanoma or non-pregnant when diagnosed with melanoma. Disease-specific survival (DSS) and disease-free survival (DFS) also served as primary outcome measures. Hazard ratio (HR) was used as the summary statistic to evaluate the impact of post-melanoma pregnancy on prognosis. The random-effects model described by DerSimonian and Laird was used to calculate summary statistics and their 95% confidence intervals. Meta-analysis results were presented on a forest plot graph. The analysis was carried out using the R software using the meta package.

## 3. Results

**Figure 1:**
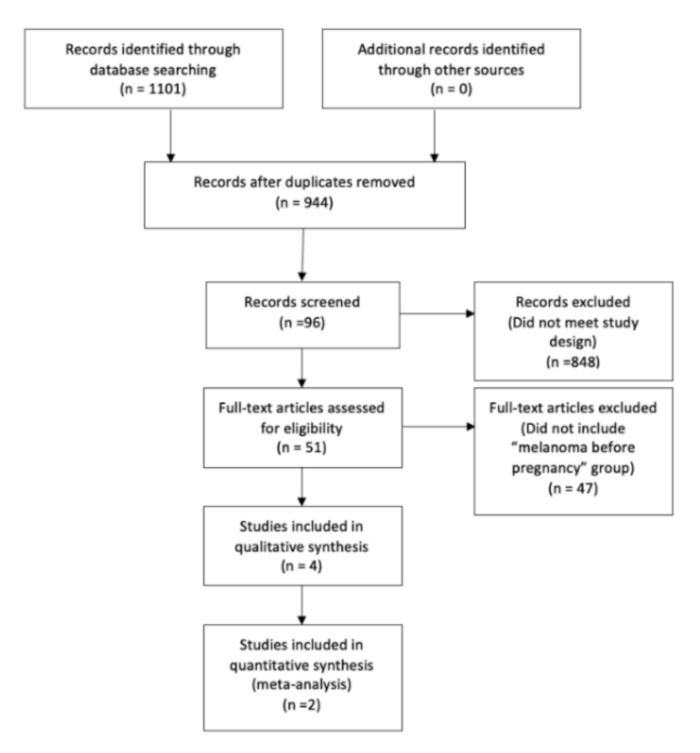
Flowchart of search strategy.

The search strategy yielded 1101 articles, of which 157 were duplicates. The titles and abstracts were then examined for eligibility, and 96 articles were included. Four reports met the inclusion criteria for the systematic review (Figure 1). All studies were retrospective non-randomised cohorts with patients diagnosed with melanoma before pregnancy: Three were population-based studies with data from statistical departments (1, 3, 4) [4,12,13,14] and one from a hospital-based population (2). None of the selected articles had the analysis of the melanoma prognosis in subsequent pregnancy as the primary outcome, so all data were secondarily extracted from the information provided.

Regarding the characteristics of the patients, those who were pregnant after melanoma were younger when they were diagnosed (range 26–29 years) than those who were not pregnant (range 36.6–40 years). Only in one study (3) [4], the time elapsed between diagnosis and pregnancy (42 months) was reflected. The follow-up period varied between 53.20 and 360 months (Table 1).

Melanoma characteristics, such as the stage of the disease at diagnosis, were not included in any of the studies. The Breslow Index was compared in 3 of the studies, but only statistically significant differences were detected in one of them, as shown in Table 2.

Finally, the most heterogeneous results were obtained in the measurement of disease prognosis, as shown in Table 3. In each study, the way of evaluating the results was different: In Lens’s [12] article, HR of global mortality is used; in Savoia’s [13], 5-year global mortality in percentage; in Stensheim’s [4] article, the crude and adjusted HR of specific mortality due to melanoma; in Vihinen’s [14], the DFS in percentage. After contacting the authors of the articles included in our systematic review to obtain homogeneous data and perform the meta-analysis, crude HR of specific death rates due to melanoma of Vihinen´s [14] became available.

**Figure 2:**
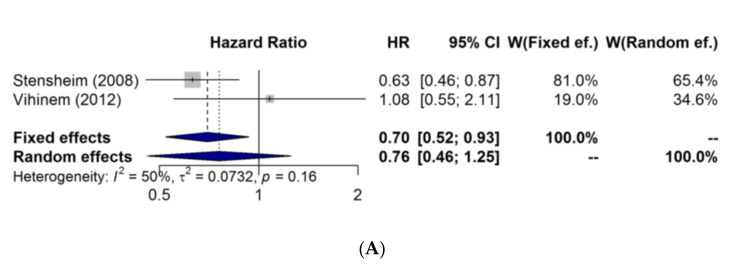

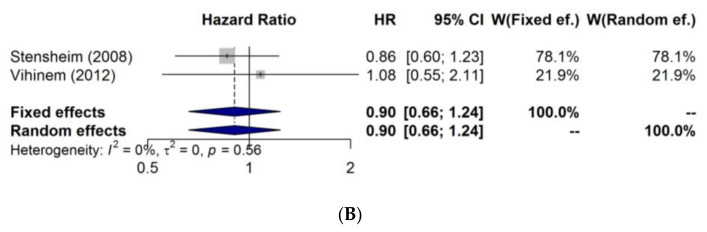
Meta-analysis of melanoma-specific mortality. (**A**) Forest plot showing pooled crude HR (Stensheim’s and Vihinen’s studies) with 95% CI for melanoma-specific death, comparing previous pregnancy or not. (**B**) Forest plot showing pooled crude HR (Vihinen’s) and adjusted HR (Stensheim’s) with 95% CI for melanoma-specific death, comparing previous pregnancy or not.

Regarding quantitative analysis, meta-analysis was pictured in two forest plots (Figure 2), combining the results of the Stensheim [4] study and those provided by Vihinen’s [14] group. In the first one, crude HR from both articles was used. As adjusted HR was not available for Vihinen’s [14] study (to control for possible differences in third variables), a second forest plot was included using the adjusted HR from Stensheim [3] and the crude HR from Vihinen [14] to show the consistency of the results.

### 3.1. Our Series

We also reviewed the medical records of patients of childbearing age who were diagnosed with cutaneous invasive melanoma in our hospital between 2008–2018 (N = 724), comparing outcomes of patients who had become pregnant and those who had not. Data were available for n = 84 women of childbearing age (35.8 ± 6.3 years, range 21–45 years) at the time of melanoma diagnosis. Of these, 11 (13.1%) had a pregnancy after melanoma diagnosis (age at pregnancy: 35.6 ± 6.3 years).

The median follow-up since melanoma diagnosis was 32.0 months (mean: 43.3 ± 35.3 months) with only one melanoma-associated death in the group of women with no post-diagnostic pregnancy and none in the group of women with a post-melanoma pregnancy. During follow-up, no deaths from other causes were recorded in either group.

**Figure 3:**
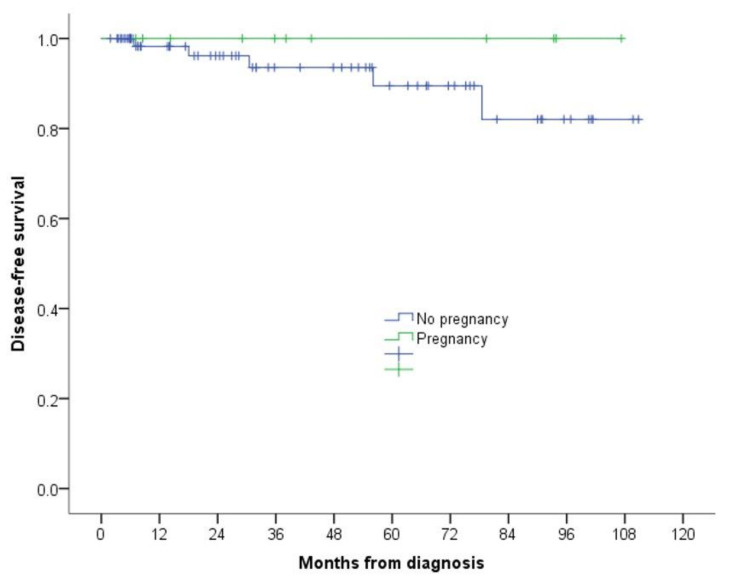
Kaplan–Meier survival curve of pregnant vs. non-pregnant women after a diagnosis of melanoma.

Regarding tumour recurrence, five (6.8%) women in the post-melanoma non-pregnant group had a relapse (one locoregional, one regional and three distant metastases), while no relapse was diagnosed in the group of women with a post-melanoma pregnancy. Disease-free survival in the non-pregnant group was 98.2% at 1 year after diagnosis, 96.2% at 2 years and 89.5% at 5 years, while it was 100% in the group of women with post-melanoma pregnancy, with no statistically significant differences (*p* = 0.304) (see Figure 3).

### 3.2. Study Limitations

Due to the relatively narrow scope of primary research or the publication bias towards articles that include patients diagnosed with melanoma during pregnancy, a scarce number of articles were finally selected. Given the small number of available databases and the heterogeneity in the design and presentation of the results, it is difficult to carry out a meta-analysis and to obtain conclusive results. Revised studies have compared global and melanoma-specific survival or disease-free survival, according to pregnancy status after diagnosis of melanoma, using traditional survival methods analysis (mainly Cox regression analysis). However, as it has been previously pointed out, Cox regression cannot totally control the confounding effect by immortal time bias when analysing an intermediate event, as is the case of pregnancy after diagnosis [15]. Immortal time bias appears when a variable, assessed post-baseline, is used in survival analysis as if it was available at baseline. It is so-called because when analysing survival, the period between melanoma diagnosis and pregnancy is “immortal” since women who reach pregnancy condition will necessarily do so alive. This follow-up time is wrongly assigned to the pregnancy group, artificially increasing the rate of death/relapse in the non-pregnancy group and decreasing, therefore, this rate in the pregnancy group. If these women had died (or relapsed) before pregnancy, they would have belonged to the non-pregnancy group. Other analysis strategies, such as including pregnancy as a time-dependent covariate in a Cox regression, or multistate models, could contribute to overcoming this bias and obtaining more valid results [16]. Given the small sample size and the few events observed, these techniques could not be used in our series.

## 4. Discussion

This study was performed with the intention of evaluating the potential influence of a subsequent pregnancy on the prognosis of a previously diagnosed melanoma and to provide the best available evidence to clinicians to help them advise women with melanoma about the potential risks of childbearing in the short to medium term.

Previous guidelines of melanoma were inconsistent in their advice to female melanoma survivors regarding future pregnancies. However, current guidelines, such as the 2019 European consensus-based interdisciplinary guideline for melanoma [17] and the 2018 American guidelines of care for the management of primary cutaneous melanoma [18], include some recommendations.

The 2019 European consensus-based interdisciplinary guidelines suggest that pregnancies in women after the diagnosis of melanoma with a favourable prognosis do not need to be deferred. However, in high-risk melanomas, the advice is to wait two years after a melanoma diagnosis since the risk of relapse is the greatest during that period, but individual factors may affect this piece of advice [17]. This guideline bases its recommendation on a narrative review, which confers a low level of evidence [18].

The 2018 American guidelines of care for the management of primary cutaneous melanoma determine that if a woman has an early-stage cutaneous melanoma (MIS or stage I), there is no need to defer subsequent pregnancies. However, in the setting of a high-risk stage II cutaneous melanoma, a 2- to 3-year delay period may be advisable considering that most recurrences may develop by this time [19]. These guidelines base their recommendations on a retrospective cohort study, which, in our opinion, entails a low level of evidence [12].

Recurrence is not always predictable, and when pregnancy is contemplated after the treatment, some factors, such as the age of the patient and the characteristics of the melanoma, need to be considered.

In terms of how hormones can affect cancer, historically, it was hypothesised that elevated oestrogen levels increase the biologic aggressiveness of cancer cells regarded as hormone-dependent, such as ovarian and breast cancer, and malignant melanoma [5,6,7,8]. There is an association between oestrogen receptor-beta expression and melanocytic lesions, with a suggestion that melanoma is a hormone-sensitive malignancy. However, the clinical relevance of oestrogen in melanoma and pregnancy-associated melanoma remains unclear [20].

Regarding the contraceptive pill and hormone replacement, there is no evidence suggesting that they confer an increased risk of melanoma [18,21]. A recent Finnish work suggests that caution should be provided for women on hormone replacement with unopposed progesterone, but most women receiving hormone replacement have combined continuous or interrupted opposition of the oestrogen with progesterone [22]. Women having oestrogen-only hormone replacement may have had hysterectomy and oophorectomy for various reasons, which may be confused with a risk factor for melanoma [17].

The results obtained in this systematic review show that there is no significant influence of a subsequent pregnancy on the prognosis of previous melanoma, but it has some limitations. None of the selected articles had as primary outcome measure the analysis of the melanoma prognosis in a subsequent pregnancy so that all data were collaterally extracted from the information provided and risk of information bias may exist. Moreover, a small number of studies with poor statistical power have been evaluated, and a meta-analysis could not be performed because of the lack of information and the heterogeneity of collected data.

Although melanoma recurrence can arise many years after initial diagnosis, the highest risk is in the first 2–3 years [20]. Therefore, some authors propose postponing pregnancy until after this immediate period following the diagnosis of melanoma.

It is recommended that patients with a history of melanoma have at least an annual full-body skin examination (TBSE), ranging from every 3 to 12 months based on the risk for recurrence and new primary melanoma [23]. Further increasing the frequency of TBSEs is not known to improve outcomes.

Other recommendations include the use of photoprotection measures, self-skin examinations and periodical dermatology visits (especially a few months before the due date).

## 5. Conclusions

Although no significant influence of a subsequent pregnancy on the prognosis of a previous melanoma has been identified in the literature reviewed due to the small number of available studies and the heterogeneity of the data included, the authors conclude that female patients should be approached with caution, and counselling should be given by known prognostic factors.

## Figures and Tables

**Table 1:** Study characteristics.

Reference	Country	Time Period	Study Design	N Cases	N Controls	Age Cases (Years)	Age Controls (Years)	Follow Up (Months)
Lens/2004 [12] (1)	Sweden	1958–1999	Retrospective cohort study	966	4567	26.5	36.60	154.80
Savoia/2007 [13] (2)	Italy	1975–2005	Retrospective cohort study	54	609	29.00	38.00	360.00
Stensheim/2008 [4] (3)	Norway	1967–2002	Retrospective cohort study	797	3949	26.00	40.00	182.40
Vihinen/2012 [14] (4)	Finland	1990–2009	Retrospective cohort study	10	55	29.00	38.30	53.20

Study characteristics.

**Table 2:** Melanoma characteristics.

Reference	Breslow (mm)		*p*
	Cases	Controls	
Lens/2004 [12] (1)	0.93	1.11	1.000
Savoia/2007 [13] (2)	1.80	1.01	0.000
Stensheim/2008 [4] (3)	missing data	missing data	missing data
Vihinen/2012 [14] (4)	1.39	1.39	missing data

**Table 3:** Disease prognosis.

Reference	Measure	Cases	Controls	All Groups	Adjusted HR	Crude HR	*p*
Lens/2004 [12] (1)	Global mortality	MD	MD	MD	0.58 (0.32–1.05) *	MD	1.000
Savoia/2007 [13] (2)	Global mortality	9.20	119.80	MD	MD	MD	MD
	Disease-free survival 5 years	72%	92%	MD	MD	MD	0.000
	Global survival 5 years	85%	98%	MD	MD	MD	0.000
Stensheim/2008 [4] (3)	Melanoma-specific death	MD	MD	MD	0.86 (0.60–1.22) **	0.63 (0.46–0.88) **	MD
Vihinen/2012 [14] (4)	Disease-free survival	54.8	29.0	46.9	MD	MD	MD

* Hazard ratio of global mortality. ** Adjusted Hazard ratio/Crude Hazard ratio for melano-ma-specific mortality. MD: Missing data.

## Appendix A

### Appendix A.1. Search Strategy

#### Appendix A.1.1. *Pubmed

(Melanoma(MH) OR Melanoma (TW) OR Melanoma, amelanotic (MH) OR Melanoma, amelanotic (TW) OR Melanoma, cutaneous malignant (TW)) AND (Pregnancy (MH) OR Pregnancy (TW) OR Pregnant women (MH) OR Pregnant women (TW) OR Maternal–Foetal Relations (MH) OR Maternal–Foetal Relations (TW) OR Infant, Newborn (MH) OR Infant, Newborn (TW)) AND (Prognosis (MH) OR Prognosis (TW) OR Neoplasm grading (MH) OR Neoplasm grading (TW) OR Neoplasm staging (MH) OR Neoplasm staging (TW) OR Treatment outcome (MH) OR Treatment outcome (TW) OR Disease-Free survival (MH) OR Disease-Free survival (TW) OR Progression-Free survival (MH) OR Progression-Free survival (TW) OR Survival (MH) OR Survival (TW) OR Mortality (MH) OR Mortality (TW) OR Survival Analysis (MH) OR Survival Analysis (TW) OR Survival rate (MH) OR Survival rate (TW) OR Kaplan–Meier estimate (MH) OR Kaplan–Meier estimate (TW) OR Demography (MH) OR Demography (TW) OR Breslow (TW) OR Breslow depth (TW) OR Breslow level (TW) OR Clark level (TW) OR TNM (TW)).

#### Appendix A.1.2. *Embase

Melanoma/OR Melanoma OR Amelanotic melanoma/OR Amelanotic melanoma OR cutaneous melanoma/OR Cutaneous melanoma) AND (Pregnancy/OR Pregnancy OR Mother foetus relationship/OR Mother foetus relationship OR Pregnant women/OR Pregnant women OR newborn/OR newborn) AND (Prognosis/OR prognosis OR Cancer grading/OR cancer grading OR Cancer staging/OR Cancer staging or Treatment outcome/OR Treatment outcome OR Disease free interval/OR Disease free interval OR Progression free survival/OR Progression free survival OR Survival/OR survival OR Mortality/OR Mortality OR Survival Analysis/OR Survival Analysis OR Survival rate/OR Survival rate OR Kaplan Meier method/OR Kaplan Meier method OR Demography/OR Demography OR Breslow OR Breslow depth OR Breslow level OR Clark level OR TNM).

#### Appendix A.1.3. *Cochrane

(Melanoma(MH) OR Melanoma OR Melanoma, amelanotic (MH) or Melanoma, amelanotic OR Nevi and melanomas (MH) OR Melanoma, cutaneous malignant) AND (Pregnancy (MH) OR Pregnancy OR Pregnant women (MH) OR pregnant women OR Maternal–Foetal Relations (MH) OR maternal–foetal relations OR Infant, Newborn (MH) or infant newborn) AND (Prognosis (MH) OR prognosis OR Neoplasm grading (MH) or neoplasm grading OR Neoplasm staging (MH) OR neoplasm staging OR Treatment outcome (MH) OR treatment outcome OR Disease-Free survival (MH) or disease-free survival OR Progression-Free survival (MH) or Progression-Free survival OR Survival (MH) OR survival OR Mortality (MH) or mortality OR Survival Analysis (MH) or Survival Analysis OR Survival rate (MH) or Survival rate OR Kaplan–Meier estimate (MH) OR Kaplan–Meier estimate OR Demography (MH) or Demography OR Breslow (TW) OR Clark level (TW)).
